# Assets and Affect in the Study of Social Capital in Rural Communities

**DOI:** 10.1111/soru.12085

**Published:** 2015-03-27

**Authors:** Martin Phillips

**Affiliations:** ^1^ Department of GeographyUniversity of LeicesterLeicesterLE1 7RHUK

## Abstract

Shucksmith (2012) has recently suggested that rural research might be refreshed by incorporating theoretical insights that have emerged through a renewal of class analysis. This article seeks to advance this proposed research agenda by exploring the concept of asset‐based class analysis and its association with the concept of social capital. The article explores connections between social capital, class analysis and understandings of community, noting how all have been associated with long running and unresolved debates. Attention is drawn to the problems of modernist legislative approaches to these debates and the value of adopting more interpretive perspectives. A distinction between ‘infrastructural’ and ‘culturalist’ interpretations of social capital is explored in relation to ‘asset‐based’ theorisations of class and culture. It is argued that an infrastructural conception of social capital might usefully be employed in association with a disaggregated conception of cultural capital that includes consideration of emotion and affect, as well as institutional, objectified and technical assets. These arguments are explored using studies of rural communities, largely within Britain.

## Introduction

‘It is too easy to accuse community of being a strawman but then not account for its popularity and social appeal. That people do yearn for and expend considerable effort in actualising community does need to be attended to and acknowledged … Despite its largely symbolic [character] … the seductions of community go beyond an abstract appeal or claim and does translate … into some small everyday acts … [that] speak of the emotional experience of community’. (Neal 2009, pp. 48–82)

‘Transformation processes that took place in contemporary societies have wrought not only profound change in the meaning of the concept of community … but also gave a new focus to the dynamics of the production of social capital’. (Carmo and Santos 2014, p. 189)

‘It is a paradox that while inequality is increasing (in rural areas as elsewhere) this has been accompanied by a declining interest in class amongst social scientists … There have been very few attempts at class analysis since 2000 … A consequence is that rural research rarely reflects the recent developments in class analysis … Specifically there is a need to research the ways in which those in similar social position … construct place and rurality, but also how these discursive and symbolic constructions are enlisted in class formation’ (Shucksmith 2012, pp. 380–389).

This article links ideas expressed above, exploring how the concept of social capital widely employed in rural community studies and, albeit to a lesser extent, in so‐called asset‐based class analysis (see Phillips 1998a, 1998b, 2007, 2011; Smith and Phillips 2001; Shucksmith 2012), might be connected to ideas of emotion and affect emerging as research foci within social and cultural studies, including those of the countryside (e.g., Edensor 2006; Carolan 2008; Heley 2010; Woods 2010; Halfacree and Rivera 2012; Hayden and Buck 2012; Phillips 2014).

Initially the article explores epistemological connections between concepts of social capital, community and assets, highlighting that these have all been subjects of long running and seemingly unresolved debates. It is argued that whilst modernist legislative approaches seek to close down such debates, they are unlikely to be successful and more interpretive perspectives should be employed, including consideration of what following Latour (1999) might be described as ‘circulating sociologies of translation’. Attention is then drawn to how concepts of community and social capital, in at least some conceptualisations, share a common focus on patterned or networked social interactions. The article explores this connection, considering its significance within debates over social capital and community, before investigating interaction‐focused conceptualisations of social capital and asset‐based theorisations of class. A series of rural studies are employed to argue that these conceptions of social capital can be employed in association with notions of institutionalised, objectified, embodied and emotional capital. The article ends by highlighting some challenges that might accompany translation of such concepts into critical studies of rural social life.

## Social capital: making sense of current debates

The concept of social capital has been widely used and debated within and beyond rural studies: indeed Van Deth (2003, p. 79) argues that its study has ‘become a minor industry in the social sciences’. Certainly rural studies have made extensive use of the concept (e.g., Falk and Kilpatrick 2000; Shucksmith 2000; Svendsen and Svendsen 2000; Chavez 2005; Lee *et al*. 2005; Carnegie Trust 2007, 2009; Svendsen and Sorensen 2007; Shortall 2008; Magnani and Struffi 2009; Carmo 2010; Sutherland and Burton 2011; Fisher 2013), although a series of criticisms and ‘legislative’ debates concerning the concept have also emerged. The term legislative refers to attempts to establish the superiority of one interpretation over others through either ‘theoretical argument and/or in relation to empirical study and/or practical relevance’ (Phillips 2009, p. 545). As outlined in Bauman (1987), and in subsequent rural studies (Murdoch and Pratt 1993; Phillips 1998a, 1998b, 2002a, 2002b, 2009; Marsden 1999), this approach was closely associated with modernism, and contrasted with so‐called ‘interpretive’ or ‘postmodern’ perspectives. Even if Bauman's distinctions are not endorsed, it is clear that attempts to delimit social capital's origins, meaning and consequences have yet to reach the epistemological ideals of clarity and widespread acceptance desired by many researchers.

In relation to origins, for example, whilst studies such as Portes (1998) locate first use of the term in the writings of Bourdieu (1980, 1983, 1984), others such as Foley and Edwards (1999) suggest that later studies, such as those of Coleman (1988a, 1988b) and Putnam (1993, 1995a, 1995b, 2000), provided more influential accounts. Putnam (2000, p. 19) himself argues that the concept of social capital was ‘independently invented at least six times over the twentieth century’, starting with Hanifan (1916) and going through unnamed ‘Canadian sociologists’ in the 1950s, Jane Jacobs in the 1960s and Glen Loury in the 1970s, before appearing in the work of Bourdieu and Coleman.

The studies identified in conceptual genealogies and the emphasis given to them clearly reflect, at least in part, preferred definitions of the concept, and it is evident that social capital represents a concept that is both ‘congested’ and ‘contested’ (Phillips 1998a, 1998b, 2002a, 2002b, 2009), with a multiplicity of conceptions and little consensus as to the most desirable. Whilst some researchers have become resigned to this situation – Szreter and Woolcock (2003, p. 5) claim that social capital appears to be destined to become ‘one of the “essentially contested concepts”’ of social science – others continue to propound the value of particular interpretations, with at least three distinct definitional strands strongly evident. First, a common theme is that social capital is constituted through social interactions and connections that take a network character. Bourdieu (1986, p. 248), for example, defined social capital as ‘the aggregate of the actual or potential resources which are linked to possession of a durable network of more or less institutionalised relationships of mutual acquaintance or recognition’. Coleman (1988a, p. 98) also emphasised interactions and connections, defining social capital as ‘the structure of relations between actors and among actors … in which others may be contacted, obligations and expectations can be safely formed, information can be shared and sanctions can be applied’. Somewhat later, Shortall (2004, p. 112) argued that the most widely accepted definition of social capital ‘is the ability to secure resources by virtue of membership in social networks or larger social structures’.

Whilst there are important differences between these definitions, they share the idea that social capital involves social interaction/connection, or what Van Deth (2003) identifies as ‘infrastructural’ perspectives on social capital. He adds that these are generally conjoined with more cultural or content based understandings, and in many instances with consequence centred interpretations. Van Deth suggests that the former define social capital in relation to cultural elements such as norms and values, with there being a range of alternative definitive propositions. These include claims that social capital involves commonality of purpose (Putnam 1995a, 1995b), norms of reciprocity (Coleman 1988a, 1988b; Svendsen and Svendsen 2000; Field 2003; Das 2004), trust (Coleman 1990; Fukuyama 1995; Putnam 1995a, 2000; Kramer *et al*. 1996; Svendsen and Svendsen 2000), civic participation (Putnam 1993, 1995a, 1995b, 2000) or learning (Kilpatrick and Bell 1998; Falk and Kilpatrick 2000). Much debate surrounding social capital may be viewed as attempts to adjudicate between particular cultural/content based interpretations in the modernist manner of promoting the superiority of one definition over others through theoretical and/or empirical argument.

The above far from exhausts identified ‘cultural’ interpretations, but highlights the complexity surrounding the concept. It illustrates, for example, how many of those involved in its definition have drawn on a range of attributes, either at different points in their writings or in proposing multi‐facetted, and in many instances integrative or more or less totalising, conceptualisations of social capital. The work of Putnam, for example, encompasses commonality of purpose, trust and civic participation, amongst other elements. Whilst these can be viewed as alternative conceptualisations, from which one or other interpretation may come to be established as more significant or fundamental than others, a common strategy is to propose integration to form aggregative conceptualisations. Lee *et al*. (2005, p. 270), for example, propose that social capital be defined, ‘[i]n the most general sense’, as formed ‘out of repeated social interactions between individuals and groups which are said to develop trust, social norms, and strengthen co‐operation and reciprocity’, a conception drawing together an infrastructural definition of social capital with substantive notions of trust, reciprocal social norms and common purpose. Another example is Falk and Kilpatrick's (2000, p. 92) differentiation of social capital into sites, processes and consequences, with interaction forming the sites of social capital, learning the process building social capital, while consequences include the formation of ‘a resource that can be stored and drawn on’.

The development of integrative/totalising concepts is a common modernistic/legislative response (Phillips 1998a, 1998b, 2002a, 2002b, 2009), although often subject to criticism. Social capital is no exception, with concerns expressed about the concept becoming so ‘elastic’ (Devine and Roberts 2003, p. 98) that it integrates the incommensurable. Woolcock (1998, p. 155), for example, argues that conceptualisations of social capital are grounded in ‘different sociological traditions’ and aggregating them together ‘risks trying to explain too much with too little’. He argues, for instance, that Coleman's conception of social capital is based on rational choice theory, whilst others develop more Durkheimian conceptions centred on social norms, or draw on Bourdieu's ideas of habitus. Combining these conceptions leads, Woolcock (1998, p. 156) argues, to a view that ‘social capital can be rational, pre‐rational, or even non‐rational’, and the associated questions of what, then ‘is it not?’ Other tensions include the degree to which social capital is a property of individuals, groups or collectives such as organisations and communities. Taken together, it is unsurprising that social capital has been described by Fine (1999, p. 9) as a ‘totally chaotic concept’.

One response to such concerns is to retreat into legislative attempts to establish the superiority of more narrowly constituted interpretations. An example is Foley and Edwards' (1999, p. 146) ‘theoretically driven review’ of 45 empirical studies undertaken in the ‘hopes of clarifying the notion of social capital … in keeping with that of Bourdieu’. Another response is to suggest that not all aspects of social capital are necessarily present in all instances, an argument that fosters identification of different forms of social capital. The most well‐known is arguably the distinction between ‘bonding’ and ‘bridging capital’ (Warren *et al*. 2001), while other differentiations include ‘integration’ and ‘linking’ (Woolcock 1998), and ‘horizontal’ and ‘vertical’ social capital (Flora and Flora 1993). These variants can be seen to rest, in part, on infrastructural and content/cultural understandings of social capital. Woolcock's distinction of integration and linking forms of capital, for example, is based on a distinction between ‘embeddedness’ that he connects to socio‐cultural relations ‘among people with common neighborhood, ethnic, religious, or familial ties’ (p. 171) and ‘autonomy’ which involve ‘social ties extending beyond … primordial [i.e., existing culturally embedded] groups’ (p. 168). Curry (2010, p. 126) similarly suggests that bonding social capital coheres around personal trust whilst bridging social capital involves ‘more distant, less interpersonal’ networks of interaction, while linking social capital involves connections to ‘unlike people in dissimilar situations’. Carmo and Santos (2014, pp. 188–189) provides a third illustration, suggesting that heightened mobility leads to shifts from bonding to bridging capital, due to declining use of ‘mutual knowledge … expressed in degrees of interpersonal trust and cultural and symbolic belonging to the same collective identity’, and increased dependence upon ‘diverse, specific social relationships and connections’ involving interactions across social groups. For Carmo and Santos, whilst bonding capital involves cultural connections involving commonality of identity/purpose/identity, trust and mutuality, bridging capital emerges from and enables extended depersonalised/de‐cultural patterns of interaction, features stressed in infrastructural conceptions of social capital.

Another response to the presence of multiple interpretations is to accept ambiguity, viewing social capital as some form of metaphor (e.g., Lee *et al*. 2005) or general heuristic (Edwards and Foley 1997, 1998). Such perspectives involve rejection of modernistic/legislative approaches and movement towards more interpretive ones. However, for some commentators such movements, and the inability to come to clear consensus as to the meaning of social capital, leaves the concept open to confusion or even an ‘empty signifier’ best avoided. Foley and Edwards (1999), for example, ask whether it is time to ‘disinvest’ in the concept of social capital, although answer a ‘qualified no’ after arguing that the concept be shorn of many of its cultural dimensions and viewed primarily in infrastructural terms, or as they describe it, as a ‘socio‐structural’ concept focused on social connections or linkages between people and/or organisations. Portes (1998, p. 1) developed a similar assessment, claiming that, ‘as shorthand for the positive consequences of sociability, social capital has a definite place in sociological theory’ but ‘excessive extensions of the concept may jeopardize its heuristic value’. Yet others suggest that there is still value in the concept, but it requires rethinking and re‐signifying (e.g., Radcliffe 2004; Holt 2008).

Similar issues surround the third definitional dimension of social capital identified by Van Deth (2003), namely its consequential dimensions. Robison *et al*. (2002, p. 2) claim that definitions of social capital often conflate ontological claims about what social capital is with consequential concerns relating to ‘what social capital can be used to achieve’. Both Van Deth and Robison, Schmid and Siles remark on how, once again, the same names appear across multiple impacts, raising similar issues of aggregative totalisation versus conceptual specificity as discussed in relation to cultural content. Shortall (2008, p. 451), for example, argues that Putnam conflates social capital with notions of social inclusion, civic engagement and participation, often viewing, for instance, ‘social capital as the same thing as civic engagement’ and yet at other points seeing ‘social capital as the cause of civic engagement, thus confusing dependent and independent variables’.

Additional concern has been raised about whether ascribed consequences necessarily flow from designated content, or whether universalistic/normative prescriptions are smuggled in. Shortall (2008), for instance, highlights how Putnam's discussion of social capital has ‘an inherent presumption’ that people should participate in social interaction and civic life. Portes (1998, 2000) similarly highlights how Coleman made implicit normative assumptions about the value of social integration, ascribing a ‘whole gamut of pathologies’ (Portes 2000, p. 2) to loss of social integration.

Utilising normative assumptions is not necessarily problematic, but within critical social science should be accompanied by self‐reflection that includes consideration as to their consequences. Shortall (2004), for example, highlights how the concept of social capital presented by Putnam, neglects state agency and places responsibility for social problems on individuals' behaviour. These characteristics are also identified by Mohan and Mohan (2002, p. 204), who argue that they act as ‘a form of revisionist neoliberalism’, enabling financial and development institutions to ignore ‘macro‐relations of power’ and ‘sidestep’ issues of the state whilst encouraging interpretations which ‘blame the victim’ (see also Harris 2001; Mayer and Rankin 2002). The ready adoption of social capital within policy and development might itself be viewed as a signal for critical reflection rather than simply celebrated as illustrative of the social relevance of the concept. Mohan and Mohan (2002, p. 205) illustrate such reflections when expressing concern that adoptions of the concept reflect its use as ‘a flag of convenience’, promoting ameliorative measures which sidestep addressing intractable, and heavily interest‐laden, issues of material inequality. Shortall (2004) raises similar issues, claiming that social capital has promoted renewal of rather romantic and naive views of rural communities.

More generally, there have been claims that the concept of social capital, particularly as elaborated by Bourdieu, creates instrumentalised/commodified views of social life. Anderson and Bell (2003, p. 240), for instance, argue that it encourages the view that everything of significance in social life can be reduced to the rational and economic, while Mohan and Mohan (2002, p. 204), drawing on Fine (1999), suggest that the concept ‘reflects the colonization of the social sciences by neoclassical economics’. More generally it has been argued that Bourdieu instrumentalised culture, interpreting the adoption of particular values and attitudes as ‘simply the pursuit of symbolic advantage’ (Cloke *et al*. 1995, p. 223). It should, however, not be assumed that this issue solely affects Bourdieu's conception of social capital. Shortall (2004, pp. 112–113), for example, notes how Putnam, whilst in one sense, gives issues such as civic engagement ‘a new injection of merit and value’, ends up reducing ‘its worth’ because it becomes discussed largely as vehicle for economic development.

The complexity and uncertainty surrounding social capital shows little sign of legislative reduction, suggesting that more interpretive responses might be in order. Such approaches might take the form of movements to heuristic or metaphorical understanding of the term, as proposed by Edwards and Foley (1997, 1998) or Lee *et al*. (2005), but there are a range of other options, including reflection on what, following Latour (1999), might be described as the ‘circulatory sociologies of translation’, or as an ‘archaeology of the category’ (Halfacree, 2001). As Phillips (2010) outlines, both terms imply that epistemological reflection needs to include consideration of the emergence and movement of concepts through the ‘hurly burly’ of social life and a range of social practices, institutions, technologies and relations. Rather more specifically, Latour suggests that concepts be viewed as produced in relationship to four ‘circulating sociologies of translation’, entitled mobilisation, autonomisation, alliance building and public representation. While the first of these relates to issues that have long been the subject of epistemological discussion, namely the relationship between concepts and objects of study, the others can also be linked to discussions of social capital.

Reference, for instance, has previously been made to social capital's widespread adoption within both academic and policy‐related discourses, features that can be viewed as indicative of autonomisation and alliance building respectively. The former refers to the extent to which concepts become incorporated into academic debates and research, whilst the latter refers to valuing by extra‐academic agencies who are willing to provide financial and other resources sufficient to sustain activities underpinning the production and movement of concepts within the other circulatory loops. As discussed in Phillips (2010), an important aspect of this is the degree to which concepts are seen to have practical policy relevance, which as previously mentioned is evidenced with social capital. Rather less attention has been paid to issues of public representation, Latour's fourth circuit. It is clear, however, that some interpretations of social capital, such as Putnam's (2000) *Bowling alone*, have a sizeable non‐academic readership: DeFilippis (2002) remarks that the article preceding this book (Putnam, 1995a), ‘exploded into the public sphere’, while Boggs (2001) claims the book elevated Putnam to celebrity status.

Academic reflections on the value of concepts rarely address these extra‐academic connections, but the implication of Latour's ‘circulatory sociologies’ is that these linkages exert significant influence even within academic debates. Fine (2002, p. 796), for example, argues that there was a decline in acknowledgements of Bourdieu as a ‘founder of social capital’ and a loss of ‘the critical aspects of his contributions’ at just the time that Putnam's work attracted public and academic attention, becoming ‘reputedly the single most cited author across the social sciences in the 1990s’. This attention has, however, not led to legislative closure on the origins, meaning and consequential significance of the concept of social capital, with many academic citations of Putnam's work being critical in tone and also, in some cases, seeking to integrate it with other conceptions, including those of Bourdieu. Even here, however, criticisms and uncertainties persist.

## Social capital, community and class assets: conceptual affinities

Given the continuing problems associated with social capital it is perhaps surprising that the concept retains its popularity, although one might argue that the epistemological dilemmas associated with it are equally evident in discussions of many other concepts, including some closely connected with it, such as community and class.

It has previously been argued that social capital is a congested and contested concept, a description equally applicable to the notion of community. Neal and Walters (2008, p. 280) characterise community as a ‘highly contested and polysemic concept’, with a range of alternative interpretations and continuing disputes between their advocates, and, indeed, about the concept's overall value. MacGregor (2001, p. 188), for instance, argued that ‘community’ is a ‘weasel word’, while Alleyne (2002, p. 608) claimed that community encompassed so many meanings that it is ‘impossible to define with any precision’.

There have indeed been repeated calls for abandonment of this concept, although community is stubbornly persistent, in part because it, like social capital, has been widely adopted by policymakers. As with social capital, this has been viewed as evidencing the terms relevance: Lees (2003, p. 78), for example, suggests that the concept of community ‘had its own renaissance’ in the 1980s, in part due to its ‘appeal to politicians and policy makers’, an attraction she links to shifts in governmentality and state/society relations (see Rose 1996; Murdoch 1997; Herbert‐Cheshire 2000; Imrie and Raco 2003; Woods and Goodwin 2003; Herbert‐Cheshire and Higgins 2004; Cheshire and Lawrence 2005). Others have expressed little but scepticism about the intentions and consequences of policy adoption of community (e.g., Levitas 2000; Fremeaux 2005; Wallace 2010, 2012).

A further parallel between the concepts of community and social capital is that both have been conjoined with macro‐scale normative/political issues. Amit and Rapport (2002), for example, argue that community studies have been drawn towards narratives of tension, conflict, exclusion, non‐place‐based interconnection, transgression and boundary mutations. They add that this is often accompanied by a loss of attention to the specificities of social life, or as Neal and Walters (2008) describe it, a hollowing out of the social from the concept of community. As with the concept of social capital, there have been calls to de‐couple the study of communities from such grand narratives, with Neal and Walters arguing for a reversal of the neglect of the social through detailed investigation of the ‘routine practices and performances of community‐making’ and ‘networks and experiences of belonging and conviviality that stem from these’ (p. 282).

Such conceptions of community have clear parallels with those of social capital, particularly infrastructural perspectives. Notions of networks of interaction have long been central to conceptualisations of community. Tőnnies' (1957) concept of Gemeinschaft, for instance, centred on notions of interaction based on kinship relations and habitual interaction stemming from common inhabitation of a territory, while many ‘classic community studies’ of the 1940s, 1950s and early 1960s expended considerable efforts in mapping patterns of interaction. Similar emphasis was evident in symbolic perspectives on community conducted in the 1980s and early 1990s (e.g., Cohen 1987; Rapport 1993), and within ‘minimalist’ community studies employing the notion of ‘local systems of interaction’ (Liepins 2000). Most recently, community studies drawing on notions of networks and sociability have also stressed patterns of interaction. Neal and Walters (2008), for example, draw on Thrift's (2005) call for recognition of the ‘forgotten infrastructure’ (p. 133) of ‘mundane activities’ (p. 135) that knot and bind cities together through affective sites in which small‐scale, ‘lighter touch urban politics’ (p. 145) generates ‘trust and familiarity’ and various mundane ‘modes of social interaction’ fostering ‘common moods’ (p. 146). This work focuses on the production of mundane practices of sociability and their everyday consequences rather than seeking to tie activities directly to ‘grand questions’,^1^ although there is also a sense that the mundane has a politics, albeit a micro‐politics or a ‘politics of small achievement’ (Lefebvre, quoted in Thrift 2005, p. 145).

Issues of political scale are also addressed in social capital analysis, with claims that this concept integrates the micro and macro (e.g., Woolcock 1998; Falk and Kilpatrick 2000). However, many criticisms of the politics of social capital arguably reflect the too hasty and direct linkage of micro configurations of the infrastructural and cultural to macro conceptions of normative and material outcomes.

Scale linkage is also an issue of concern within class analysis. As noted earlier, Shucksmith (2012) suggests that there has been relatively little rural class analysis since the turn of the millennium, despite a ‘reinvigoration’ of class analysis across the wider social sciences (see also Phillips 2011). Amongst the issues addressed within this ‘renewal of class’ (Crompton *et al*. 2000; Bottero 2004) was consideration of scale, there being calls for more micro‐scale analysis and studies that link or transcend the micro‐macro distinction. Savage *et al*. (2005, p. 31), for example, argue:

‘since the 1980s defenders of class analysis have shifted their foundations away from … [a] “macro” emphasis on the division of labour towards a more “micro” interest in how the effects of class are produced through individual actions drawing variously on “assets” … “capitals”,… or “resources” … These concepts, which we term collectively “CARs” (capitals, assets and resources), litter recent works on the sociology of stratification’.

They suggest that CARs ‘spans the macro‐micro divide’ (p. 43), although add: ‘To date, the promise of CARs based approaches to offer a clear theoretical foundation for a revived class analysis has not yet been fulfilled’.

CARs, or what Savage and Butler (1995) previously described as ‘assets‐based class analysis’, is hence programmatic rather than fully developed. Despite this, Savage *et al*. (2005, p. 43) suggest that there are ‘pointers to how it might be achieved’, with both they, and Shucksmith (2012), being drawn to Bourdieu's elaboration of social and cultural capital. For Savage, Warde and Devine, such conceptions, along with a range of other forms of capital, provide the basis for assets‐based theorisations of social class that sees class as an emergent effect of the structuring of many fields of CARs.

One of the first studies explicitly encompassing such an approach is Savage *et al*.'s (1992) *Property, bureaucracy and power* which draws on Bourdieu (1984) and Wright (1985) to suggest that class positions are formed through combinations of economic and organisational assets and culturally credentialed skills. A subsequent study, *Culture*, *Class*, *Distinction* (Bennett *et al*. 2009) develops the arguments further, focusing attention on the concept of cultural capital which had informed the earlier discussion of skills and culture. In particular, they suggest that cultural capital needs to be disaggregated, being viewed in effect as an ‘umbrella term for a range of different cultural assets’ (p. 36), each of which should be assessed independently so as ‘to better assess the extent of their relative scope and effectivity’ rather than, as Bourdieu did, fold them ‘into one another in relations of necessary connection’ (p. 29).

There are clear parallels here with debates over social capital and the degree to which various constituent elements should be integrated to provide a totalising concept or whether such an approach ignores the specificities of each element. Bennett *et al*. (2009, p. 29) argue for disaggregation, pointing to a distinction made by Bourdieu (1989) between what they characterise as ‘subtypes of cultural capital’, namely institutional, objective and embodied capitals. They admit that the ‘exact provenance and content’ of these forms of cultural capital ‘is difficult to pin down’, although suggest that institutional capital refers to social status or value derived from qualifications or ‘credentials’, objective capital relates to the possession of objects and to ‘judgements of taste’ associated with them, whilst the notion of embodied cultural capital,

‘seems to refer sometimes to resources that others call human capital – skills and competences, cognitive and manual, which when deployed may be sold or gifted to others. On other occasions it seems to refer to bodily hexis, to accent, posture and demeanour; to the appearance and presentation of the body to others’. (p. 153)

They also suggest that Bourdieu's distinction between embodied, objectified and institutionalised cultural capital is ‘valuable but restrictive, and needs to be supplemented’ by consideration of a range of additional forms of cultural capital such as ‘technical, emotional, national and subcultural’ (p. 258).

Bennett *et al*.'s arguments could be seen as a further instance of the ‘plethora of capitals’ which Baron and Hannan (1994, pp. 1122–1124) complain have emerged over recent years, whereby social scientists ‘have begun referring to virtually every feature of social life as a form of capital’. As discussed previously, this risks social and cultural capital becoming, at best, chaotic concepts, and at worst empty signifiers. Indeed, within rural studies and elsewhere there is arguably a tendency to conflate and/or use the two concepts interchangeably. However, Bennett *et al*. seek to integrate the identification of capitals into broader frameworks and empirical analysis, including asset or CAR based analysis of social class. The next section explores the extent to which the distinctions between institutional, objectified, embodied, technical and emotional capital connect to the concept of social capital, rural class analysis and, at least potentially, issues highlighted in the emotional and affective turns impacting rural studies (e.g., Anderson and Smith 2001; Thien 2005; Clough and Haley 2007; Davidson, Bondi and Smith 2007; Pile 2010; Pini, *et al*. 2010a, 2010b; Phillips 2014).

## Forms of cultural capital and social capital: traces within British rural studies

**Figure 1:**
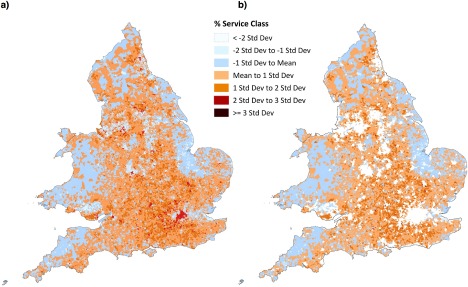
The relative significance of the service class in (a) England and Wales and (b) rural areas in England and Wales, 2011 Sources: Derived from Offce for National Statistics, 2011 Census: Aggregate data (England and Wales) [computer file], UK Data Service Census Support (Downloaded from: http://infuse.mimas.ac.uk. Information licensed under the terms of the Open Government Licence [http://www.nationalarchives.gov.uk/doc/open‐government‐licence/version/2]); Digitised Boundary Data (England and Wales) [computer file], UK Data Service Census Support (Downloaded from: http://edina.ac.uk/census); Rural‐urban classification (2011) of output areas (2011) E + W (Downloaded from: https://geoportal.statistics.gov.uk).

As Shucksmith (2012, p. 388) remarks, the late twentieth century saw ‘a flurry of research on the role of class in rural areas’. A major stimulus to this was the rise of political economy approaches to rural studies (see Cloke 1989; Phillips 1998c, 1998d), with particular attention paid in British studies to the growth of a ‘service‐class’ composed of managers and professionals (e.g., Cloke and Thrift 1987, 1990; Thrift 1987a, 1987b). Work placed great emphasis on skills and credentials, claiming these had become prominent within capitalist divisions of labour and a profound influence on the social composition of rural areas because members of the service class, whose members were seen to have the organisational and other skills required within emerging forms of post‐industrial production, were attracted to living in the countryside (e.g., Cloke and Thrift 1987, 1990). Analysis of the 2011 Census broadly supports this argument (Figure 1), although also suggests that the arguments of Hoggart (1998) and Phillips (2007) concerning the presence of other middle class groups in the British countryside is of continuing significance (Supporting Information Figure S1).^2^

Discussions of the service class and significance of organisational and other skills have been connected to notions of cultural capital, not least by Savage *et al*. (1992) who connect the class analyses of John Goldthorpe and Erik Wright with the concept of cultural capital as elaborated by Bourdieu (see Phillips 2007). Amongst their arguments is that skills and credentials are defined in relationship to cultural fields that work to value ‘certain types of activities … more than others’ (Savage *et al*. 1992, p. 16). Such claims were developed in Bennett *et al*. (2009, p. 258), in part through reference to Bourdieu's notion of ‘technical capital’, described as ‘cultural resources, or skills,… that allow individuals to accumulate potentials and capacities’ and which are ‘marketable’,^3^ and the notion of ‘institutional capital’, which as noted earlier, relates to qualifications or credentials.

**Figure 2:**
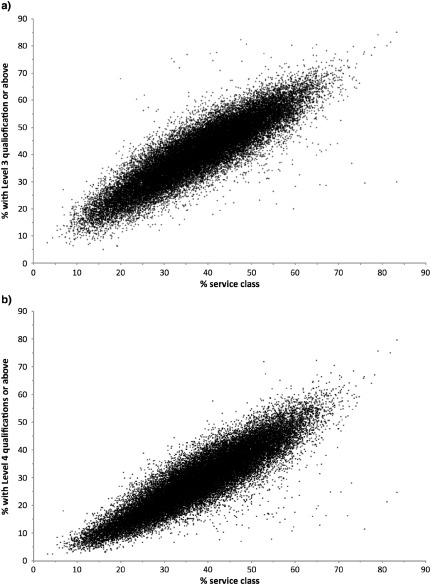
Proportion of residents with (a) Level 3 and (b) Level 4 or above qualifications against percentage of service class population, rural output areas in England and Wales Sources: Derived from Offce for National Statistics, 2011 Census: Aggregate data (England and Wales) [computer file], UK Data Service Census Support (Downloaded from: http://infuse.mimas.ac.uk. Information licensed under the terms of the Open Government Licence [http://www.nationalarchives.gov.uk/doc/open‐government‐licence/version/2]); Rural‐urban classification (2011) of output areas (2011) E + W (Downloaded from: https://geoportal.statistics.gov.uk).

Notions of technical and institutional capital are potentially relevant to rural class analysis. Phillips (2011), for example, highlighted evidence of educational attainment being associated with social class differences amongst rural residents. Analysis of the 2011 Census provides further support, with, for example, the proportion of people who have Level 3 qualifications or above increasing broadly in line with the proportion of an area's population in service class occupations (see Figure 2 and Supporting Information Figure S2).

Connected to arguments about the significance of skill and qualifications within service class formation, were claims that these groups preferred to reside in areas with good access to cultural facilities (e.g., Cloke and Thrift 1987). Rural class analyses of the late 1980s and 1990s often framed these arguments in terms of a heightened signification of consumption within class formation and migrational decision‐making (e.g., Cloke and Thrift 1987, 1990; Thrift 1987a, 1987b; Shucksmith 1990; Marsden *et al*. 1990; Phillips 1993), with there being reference to notions of cultural capital as outlined by Bourdieu, albeit rarely developed to any significant extent. This may have reflected reservations about aspects of Bourdieu's conception of cultural capital. Cloke, Phillips and Thrift (1995, p. 223), for example, stated that they had ‘chosen elsewhere’ than Bourdieu for inspiration of how to ‘examine the relationship between class and culture’, because they felt ‘Bourdieu's analysis radically downplays cultural complexity’ as well as understating the role of agency and ‘tactics’ in the formation of culture (see also Phillips 1998a, 1998b, 2001, 2002a, 2002b). However, Bennett *et al*.'s disaggregation of cultural capital goes someway to adding complexity into the concept, and subsequent studies have sought to further contextualise the analysis of cultural capital (e.g., Prieur and Savage 2011; Meuleman and Savage 2013; see also Lamont 1992, 2000). Drawing on this disaggregation, much of the discussion of class and cultural capital conducted in rural class analysis of the 1980s and 1990s might be reframed as analyses of objectified cultural capital. As defined by Bennett *et al*. (2009, p. 29), this form of cultural capital relates to ‘possessions and to the judgements of taste associated with their acquisition’.

As noted in Phillips (1998a, p. 421), rural based analyses of class and cultural capital often focused on rural housing, widely considered as a mechanism for ‘positional consumption’ in that its ownership was ‘both dependent upon, and in turn help[s] … to create, people's position in society’. Work such as Sturzaker and Shucksmith (2011) and Shucksmith (2011) highlights continuing discrepancies between rural and urban house prices in the UK, suggesting that rural housing still acts as an important form of objectified cultural capital. However, one implication of Bennett *et al*.'s theorisation of this capital is that it can involve a whole range of commodities, a point made by Phillips (2002b, p. 288) who remarked that positional consumption can be ‘applied to a number of “rural objects”’ other than housing, including ‘landscapes … leisure activities … consumer goods … and participation in social institutions’. Drawing on Lamont's (1992) work on boundary drawing, he further argues that social positions can be inscribed, and devalorised, in a variety of ways, including through the use of cultural and moral, as well as socioeconomic, forms of classification (see Phillips 1998b, 2001, 2002b; and Heley 2010).

Bourdieu's analysis of objectified, and indeed other forms of cultural capital, draws heavily on ideas of cultural classification, such as notions of ‘legitimate’ and ‘illegitimate'/‘alternative'/‘popular’ cultures or ‘high’ and ‘low'/‘low‐brow’ cultures. People such as Bennett *et al*. (2009), Lamont (1992, 2000) and Sayer (2005, 2010) have all argued that such systems of classification have less value in understanding the dynamics of culture and class than assumed by Bourdieu, although all suggest that they are of some significance. Bennett *et al*. (2009, p. 49), for example, suggest that the ‘prime cultural division in contemporary Britain’ does not lie ‘between “high” and “popular” culture’ but between those who are engaged in cultural activities of all kinds and those who are not. However, members of what they describe as a professional‐executive class, composed of professionals and employers in large establishments, are, as a group, more regular attendees at ‘high culture’ institutions such as opera, cinema, theatre, museums and art galleries.

There has been few explicit explorations of systems of cultural classification in the countryside, although Phillips (1998b, 2001) has suggested that some rural gentrifiers make use of ‘high cultural’ markers associated with particular commodities and places, Edensor (2006) and Heley (2010) have emphasised how activities such as hunting and shooting have acted as markers of identity in the UK countryside, and Phillips (2011) has highlighted rural residents' differential involvements in ‘high cultural’ activities such as watching opera, theatre and films at the cinema (Table 1).

**Table 1:** Attendance at cultural events by residents of 5 villages in Leicestershire and Warwickshire

Cultural activity and frequency of attendance	Percentage of residents by social class, as indicated by classification of Goldthorpe, Llewellyn and Payne (1980)							
	I	II	III	IV	V	VI	VII	Total
Cinema								
Monthly or more	19.4	11.7	15.8	7.1	0.0	0.0	4.3	12.2
Monthly to 6 monthly	9.7	3.3	2.6	0.0	0.0	0.0	0.0	3.3
Six monthly or less	32.3	26.7	23.7	7.1	10.0	33.3	17.4	24.3
Annually or less	19.4	31.7	21.1	50.0	40.0	16.7	17.4	25.4
Never	19.4	26.7	36.8	35.7	50.0	50.0	60.9	34.8
Theatre								
Monthly or more	9.7	16.7	17.1	21.4	10.0	0.0	4.8	13.2
Monthly to 6 monthly	9.7	1.7	4.9	0.0	0.0	0.0	0.0	3.3
Six monthly or less	38.7	30.0	19.5	14.3	10.0	50.0	23.8	28.0
Annually or less	32.3	36.7	24.4	35.7	40.0	0.0	4.8	26.4
Never	9.7	15.0	34.1	28.6	40.0	50.0	66.7	29.1
Opera								
Monthly or more	0.0	1.7	4.9	0.0	0.0	0.0	4.3	2.2
Monthly to 6 monthly	0.0	0.0	0.0	0.0	0.0	0.0	0.0	0.0
Six monthly or less	6.5	5.0	2.4	7.1	0.0	0.0	0.0	3.8
Annually or less	29.0	13.3	12.2	14.3	10.0	16.7	4.3	14.8
Never	64.5	80.0	80.5	78.6	90.0	83.3	91.3	79.2

Whilst social differentiation in objectified cultural capital exists, Bennett *et al*. (2009) suggests it is less significant than assumed by Bourdieu, at least in a British context. They suggest, for example, that whilst professional‐executive class members generally engage with activities ‘high up’ on cultural hierarchies to an extent greater than other classes, such participation is ‘not by a long chalk’ (p. 253) undertaken by all members of this class. They further argue that whilst participation in high cultural activities can be linked into class formation for elite elements of this class, ‘where it oils the wheels of social connections’ (Bennett *et al*. 2009, p. 253) and/or assists in the development of technical knowledges, even within this class it is widely accorded little value.

These arguments are significant both with respect to cultural and social capital. In relation to the former, they suggest that an emphasis on cultural hierarchy is place, and indeed time, specific, an argument supported by the cross‐national studies of Lamont (1992, 2000) who suggested that cultural boundaries were strongly drawn upon in assessments of people's worth amongst French middle class men, and indeed amongst working class men as well, but were less significant in the boundary work done by American middle and working class men, who tended to make greater use of socio‐economic distinctions. This does not mean that culture was insignificant within class formation, but rather that forms other than objectified culture can be significant.

Amongst these other forms of capital identified by Bennett *et al*. (2009) was embodied capital. As discussed earlier, they suggest Bourdieu's writings on this form of capital often present it in a manner akin to technical capital, although in instances it appears to refer to embodied dispositions, such as posture, appearance and ways of speaking, moving and acting. As such it can be seen to connect to Bourdieu's concept of ‘habitus’, understood as ‘ways of doing and being which social subjects acquire … through lived “practice”’ (Lovell 2001, p. 17), and with notions of social capital centred on sociability and embodiment. Holt (2008, p. 242), for example, suggests that social capital be rethought as embodied capital encompassing ‘informal (along with formal), everyday (although not necessarily copresent) emotionally painful and gratifying social relationships that make up the gritty lived “reality” of social life’, a perspective she connects to more conventional critical concerns, such as ‘how such relationships variously confer capitals and thus (re)produce or transform broader socio‐spatial axes of inequality’. In a sense, Holt proposes a return to an infrastructural view of social capital, albeit focused on embodied inter‐connection rather than abstracted ones as implied, for instance, by a concern with elucidating densities of inter‐connection.

Holt adds that such a view ‘resonates strongly’ with concerns within contemporary human geography relating to ‘the beyond consciousness, the reflexive and the Affectual realm’ (p. 233), notions that as mentioned earlier, have begun to influence rural studies. Heley (2010, p. 3), however, notes that such studies, at best, ‘make only fleeting references to … class’ and Holt herself argues that,

‘performativity/non‐representational theories tend to … shy away from explicitly exploring how such identity performances are interconnected with, (re)produce and can transform, broader scale “patterns” of material sociospatial inequality’. (p. 237)

She adds that in her view, the mundane yet complex practices of everyday interactions should be ‘read’ against some form of ‘sociostructural framework’ in order to evaluate ‘how everyday performances (re)produce or transform socio‐spatial expressions of inequality operating at a variety of interconnected spatial scales’ (pp. 237–238).

One illustration Holt gives is to suggest that recursive relationships exist between social and cultural capital. Making use of the distinction between institutionalised, objectified and embodied cultural capital, she suggests that particular forms of institutional and objectified capital can ‘open up spaces’ (p. 232) for social interactions. Clear rural illustrations can be identified. Milbourne (2003) and Heley (2010), for example, respectively highlight how participation in hunting and shooting acts as vehicles for social interaction as well as performing rural social identities, whilst Neal (2009) explores the role of Young Farmers Clubs and Women's Institutes as places of conviviality. Four decades earlier, Bourdieu himself began detailing the social interactions enacted in rural settlements in the French Pyrenees (e.g., Bourdieu 1962a, 1962b, 1989, 2008), highlighting in particular how dance halls provided venues for highly embodied, and in many instances quite awkward, social performances.

As Bourdieu's studies of dance halls emphasised, embodied cultural capital can impact on social capital as people come, or fail, to take in ‘the dispositions and manners that facilitate the types of appropriate sociability’ (Holt 2008, p. 232), an argument illustrated by Heley (2010) and Edensor (2006) who highlight the objects and ways of acting associated with shooting and hunting. More generally, Prieur and Savage (2011, p. 576) remark that the structuring of tastes,

‘inclines people to choose as friends and partners people who have the same tastes (and opinions) as themselves, as well as to settle in neighbourhoods with people who resemble themselves, and to put their children in schools with children from the same background as themselves’.

As previously discussed, Bennett *et al*. consider that ‘legitimated’ objectified cultural capital can act, in some instances, to facilitate social connection. However, not only can this form of cultural capital facilitate the formation of social capital but there may also be connections to other, more economic and political forms of capital, a point explicitly recognised in many accounts of social capital. Portes (1998, p. 9), for example, argues that literature reviews reveal that social capital is ‘frequently invoked … as an explanation of access to employment, mobility through occupational ladders, and entrepreneurial success’. Holt (2008) also highlights the significance of emotion within social capital, an argument also made by Reay (2000, 2004), who seeks to extend Bourdieu's ‘conceptual framework’ to encompass the notion of emotional capital. She notes how this term was used by Nowotny (1981), who viewed it as a variant form of social capital, although Reay connects it to cultural capital as well as to ideas of habitus and embodied capital. Reay (2000) develops her arguments by viewing families as institutions through which embodied and, in some cases, other forms of cultural capital are produced and conveyed, which in turn influences the accumulation of more institutional forms of cultural capital associated with school education. As Prieur and Savage (2004, p. 568) note, Bourdieu initially developed his conceptions of cultural capital ‘to explain the higher success rates for the children of educated parents in educational attainment’, so in a sense Reay's research marks a return to well established argument. However, whilst Probyn (2004), Silva (2007) and Sayer (2010) all argue that Bourdieu pays little attention to emotions, Reay emphasises the significance of these, claiming that feelings of anxiety, anger, guilt and embarrassment were prominent in discussions of schooling by both working‐ and middle‐class mothers, although the subjects and effects of these emotions could differ widely.

Reay develops her arguments through the notion of emotional capital, although as Zembylas (2007, p. 447) notes, others have emphasised emotional dimensions of Bourdieu's analysis of habitus through a range of other concepts, including Williams' (1977) notion of ‘structures of feeling’. Reay employs the term ‘more as a heuristic device … than as an overarching conceptual frame’, explicitly remarking that the concept needs ‘refining both theoretically and empirically’. This call has been heeded to a degree, with a series of empirical and theoretical studies of emotional capital emerging over subsequent years (e.g., Reay 2004; Colley 2006; Gillies 2006; Illouz 2007; Zembylas 2007; Bennett *et al*. 2009; Nixon 2011; Feeney and Lemay 2012; Hutchison 2012), although Reay's judgement about the need for further conceptual refinement seems relevant a decade on. There is, for instance, a dearth of clear descriptions as to the precise meaning of the term, as well as significant differences in interpretation. For example, while Reay's use of the term encompassed negative feelings such as anxiety, embarrassment and anger, Feeney and Lemay (2012, pp. 1004–1005) explicitly reject this, arguing that emotional capital be viewed as ‘an accumulated stock of “relationship wealth” made up of a set of positive, shared emotional experiences that constitute a resource inherent to a particular relationship’ and that whilst such experiences ‘can emerge from negative life events’, experiences that ‘are shared but not positive’ do not constitute emotional capital. Such arguments seem unnecessarily restrictive, particularly given Reay's earlier demonstration of the significance of potentially destructive emotions in motivating action. Sayer's (2005) concept of ‘emotional commitment’ might be more appropriate as it holds scope for encompassing negative as well as positive feelings. Feeney and Lemay do, however, make the important point that emotional capital involves not simply cognitive emotions but also involve relations that are, affectual, or beyond the bounds of cognition (see also Phillips 2014).

Feeney and Lemay, and Reay (2000, 2004), also suggest that there are complex relationships between emotional and other forms of capital. Reay, for example, highlights that while emotional commitments of parents to their children's education is often interpreted as linked to the accumulation of educational qualifications, they are often impacted by ‘economic security and social status’ (Reay 2000, p. 582), an argument of wider relevance. Studies of service sector occupations, for example, have long recognised that employees may require embodied skills related to ‘looks, personalities and emotions’ (Leidner 1991, p. 156), an issue picked up in some studies employing the concept of emotional capital. Illouz (2007, p. 66), for example, argues that ‘emotional competence has … become a formal criterion for recruiting and promoting people’, and also that emotion has become the focus of an emerging set of professions concerned with ‘the management of emotions – especially of the new middle class’. Whilst Illouz talks about emotions as formal criteria of appointment, it is evident that emotions are complexly related to cultural qualifications and constructions of identity: as Silva (2000, p. 3) notes, emotions are incorporated into classificatory systems, being widely contrasted and devalued against other facets of social life, such as thought and action, as well as differentiated and evaluated against one another, and associated and/or disassociated with particular behaviours, types of people, situations or places.

Recognition of emotion has become an important strand of ‘renewed class analysis’ (Gillies 2006), as well as, as noted previously, an emergent focus of rural studies. They have, however, yet to become a subject of sustained investigation within rural class analysis, although there are pointers suggesting scope for such studies. Phillips (2011, p. 45), for example, argues that Abram's (1998, p. 373) claim that rural studies has an ‘obsession with class’, ‘could be interrogated from an emotional direction rather than in relation to discourses of class and classlessness’ and that Sayer's (2005) distinction between investment and commitment could be used to explore Cloke *et al*.'s (1998, p. 179) suggestion that rural in‐migrants are ‘investing not only socially and economically but also culturally and psychologically’. Sayer, like Cloke, Phillips and Thrift, has reservations about the instrumentalisation of action implied in many aspects of Bourdieu's work, including his notions of cultural capital, suggesting that whilst actors at times clearly undertake quite strategic, self‐interest focused, action, they also often commit to actions ‘not merely for the reward but because they come to see … [things] as valuable in themselves, sometimes regardless of any benefit to themselves’ (Sayer 2005, p. 40).

As discussed in Phillips (2014, p. 60), the study of emotion and affect has been seen to require the adoption of new, non‐ or more‐than representational methodologies, but may also involve the re‐working of ‘mainstay’ qualitative methods that both allow people to convey ‘conscious emotions’ but may also reveal ‘traces of pre‐ and non‐cognitive modalities of affect … in ways which may be far from mimetic’. The value of such re‐workings can be illustrated by an examination of ‘classic’ rural community studies, which can be seen to contain significant, if hitherto unacknowledged, references to emotion and affect in the formation of community life (Phillips and Walkerdine, forthcoming). Here for example, are two extracts from Williams' Gosforth study:

‘attitudes towards certain parishioners, both as individuals and groups, tend to vary along well defined lines … There were men who were “a different type from the Village” and others were “people who won't acknowledge you”. There were “better class folk who make you *feel* awkward” and “people who are different because of *the way they carry on*”’ (Williams, 1956, p. 87, emphasis added).

‘The subordination of farmers' children was said by many informants to have been so marked until very recently that several farmers' sons in the district had killed themselves rather than submit to further repression. One of the few suicides in Gosforth was described as due to this type of treatment. Apparently the man concerned was friendly with a group of farmers' sons and farm labourers who were generously supplied with money, and his *constant failure to emulate their behaviour*, together with their remarks and taunts, *drove him to this extreme act*. It is, of course, difficult to judge how much truth there is in this account, but there can be no doubt that the present occupiers of farmsteads were completely dependent on their parents during their youth, and often for many years after they had become adults … The younger members of farm families accept this state of dependence *without any overt sign of resentment*. They are … accustomed to paternal dominance until they marry; and since their position of dependence lasts often until they are over 30 years of age they are frequently *little removed psychologically from a state of adolescence* when they assume the responsibility of a holding’. (pp. 42–44, emphasis added)

Such quotes can be interpreted as illustrative of patterns of interaction linked to constructions of cultural identities – of villagers and non‐villagers, of upper and lower class, of farm families – and to strategies of economic accumulation. They can also be linked to notions of social capital and its convertibility into cultural and economic capital. However, the emphasis added highlights emotional/affective dimensions to this material that have been largely ignored in existing interpretations of this work, and indeed arguably from rural community studies more generally. The account of Williams, for example, resonates with recent studies of the emotional demands of agriculture (e.g., Bennett 2004; Ramirez‐Ferrero 2005; Price and Evans 2009), and with Bourdieu's own ‘phenomenological analysis’ of the ‘emotional life’ of bachelors in rural France (Bourdieu 2008, p. 2; see also Bourdieu 1962a, 1962b). It can also be seen to demonstrates the clear concern with class relations and differences that Savage (2010, p. 139) claims reflects Williams' own feelings of ‘outsiderness’ in the village he was studying, as well as earlier experiences of ‘the manners’ of London students in a Welsh University. Williams' emphases on class and the emotional/affective dimensions of rural life, were, however, largely subsumed by other foci including network relations of interaction, relations between land and the fate of community relations and identities (see Phillips 1998d). There has, however, been recent recognition of the significance of emotional relations within rural class analysis, albeit within Australia, where work has explored emotions associated with moral worth and disgust (Bryant and Pini 2009; Pini, Mayes and McDonald 2010a; Pini, Rice and McDonald 2010b; see also Phillips 1998a, 1998b; Henderson 2005), as well as feelings of anger, anxiety, bewilderment, betrayal, resentment and fear associated with the closure of a rural mineral mine (Pini, Mayes and McDonald 2010a; Pini, Rice and McDonald 2010b). Whilst the latter study focused on a particular acute situation, it is argued that the responses resonated strongly with ‘the work of contemporary class scholars’ such as Skeggs (1997, 2004) and Sayer (2005).

## Conclusion

This article has reviewed debates over social capital, exploring connections and parallels to discussions of community and asset‐based class analysis. It has argued that debates over social capital exhibit many modernist/legislative characteristics as identified by Bauman (1987), although questions remain about the overall validity of the term. Devine and Roberts (2003, p. 94) suggest that social capital be ‘explored at different levels of “abstraction”’, rather than discussed in terms of alternative definitions. This proposal draws on notions of critical realism which links abstractions through notions of commensurability, but there may well be scope for more ‘complementary perspectives’ (Phillips 1998a, 1998b, 2002a, 2002b) which draw together incommensurable conceptions in supplemental ways. This article suggests that van Deth's (2003) distinction between infrastructural and cultural conceptions of social capital is a potentially useful one, particularly when combined with notions of embodiment and disaggregated notions of cultural capital as outlined by Bennett *et al*. This latter perspective brings the concept of social capital into an articulation with ideas emerging from asset‐based class analysis as well as recent moves to recognise more affective and emotional dimensions of social life.

**Figure 3:**
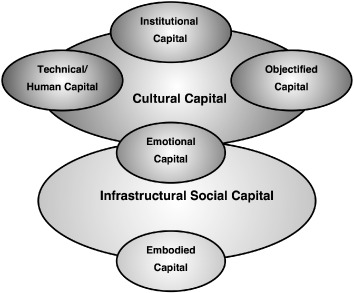
Infrastructural social capital and forms of cultural capital.

Figure 3 is a diagrammatic representation of these arguments, developed in this article with particular reference to British rural studies of social capital and class, although also making recourse to some studies of rural Australia and France as well as more general sociological literatures. Attention was drawn to the emphasis placed on skills and credentials in conceptions of the service class which stimulated rural class analyses in the late 1980s and 1990s, it being argued that such research illustrates the significance of technical, institutional and objectified forms of cultural capital. Connections between these forms of cultural capital and social interaction, or infrastructural social capital, were then highlighted. Whilst the latter can be conceptualised as the density of inter‐connections between people, embodied perspectives highlight connections to emotional and affective relations/tensions created both as part of practices of embodied interaction and in association with the cultural content of these interactions. Hence relationships of technical, institutional and objectified capital both require embodied interaction and sustain/create affective/emotional connections and commitments. Consequently, social capital can be seen to both involve embodied and emotional capital, and to be recursively involved in, and draw from, technical, institutionalised and objectified forms of capital.

Mention has been made to Shucksmith's (2012) call for engagement by rural studies with theoretical arguments from a renewed class analysis, and in a sense this article is a response to this. However, Shucksmith (2012, p. 392) also highlights the need to develop such research not simply as discursive and symbolic constructions ‘on paper’ but to detail connections to processes of class formation within particular ‘historically embedded and locally specific practices’. The recent re‐publications of some of Bourdieu's early rural research might itself be seen to provide some inspiration or model for such examination, but also provide cautionary lessons as well, not least with respect to the difficulties of capturing the embodied, affectual and emotional dimensions of rural life and creating an effective critique of the class relations that infuse these:

‘nothing, except perhaps the pent‐up tenderness of the description of the ball, evokes the emotional atmosphere in which my fieldwork was conducted … I feel the sense of something of like a betrayal – which has led me to refuse to this day any republication of texts whose appearance in scholarly journals with small readership had protected them against ill‐intentioned or voyeuristic readings’ (Bourdieu 2008, p. 3).

## Supporting information


**Figure S1.** The relative significance of the middle class in (a) England and Wales and (b) rural areas in England and Wales, 2011. Sources: Derived from Office for National Statistics, 2011 Census: Aggregate data (England and Wales) [computer file], UK Data Service Census Support (Downloaded from: http://infuse.mimas.ac.uk. Information licensed under the terms of the Open Government Licence [http://www.nationalarchives.gov.uk/doc/open‐government‐licence/version/2]); Digitised Boundary Data (England and Wales) [computer file], UK Data Service Census Support (Downloaded from: http://edina.ac.uk/census); Rural‐urban classification (2011) of output areas (2011) E + W (Downloaded from: https://geoportal.statistics.gov.uk).

**Figure S2.** Proportion of residents with Level 4 qualification within rural output areas in England and Wales. Sources: Derived from Office for National Statistics, 2011 Census: Aggregate data (England and Wales) [computer file], UK Data Service Census Support (Downloaded from: http://infuse.mimas.ac.uk. Information licensed under the terms of the Open Government Licence [http://www.nationalarchives.gov.uk/doc/open‐government‐licence/version/2]); Digitised Boundary Data (England and Wales) [computer file], UK Data Service Census Support (Downloaded from: http://edina.ac.uk/census); Rural‐urban classification (2011) of output areas (2011) E + W (Downloaded from: https://geoportal.statistics.gov.uk).

## Notes

1 Significantly, Thrift (2005, p. 145) argues that much of the mundane activities he is describing can ‘easily be confused with other agendas, for example in attempts around the world to build “social capital”’ but resists making such connections, in part because he views these social interactions as being ‘more’ (p. 138) than implied by this concept.

2 This article includes two Supporting Information Figures which are accessible on‐line although do not appear on the printed version of the article.

3 This description of technical capital not only takes the concept close to that of human capital, as acknowledged by Bennett *et al*. (2009), but also arguably close to Wright's (1985) conception of skill assets that Savage *et al*. (1992) were critical of.
