# Supplementary figures and images for: Assets and Affect in the Study of Social Capital in Rural Communities
Source: Sociol Ruralis. 2015 Mar 27;56(2):220–47. doi: 10.1111/soru.12085 (PMC4975700; doi:10.1111/soru.12085)

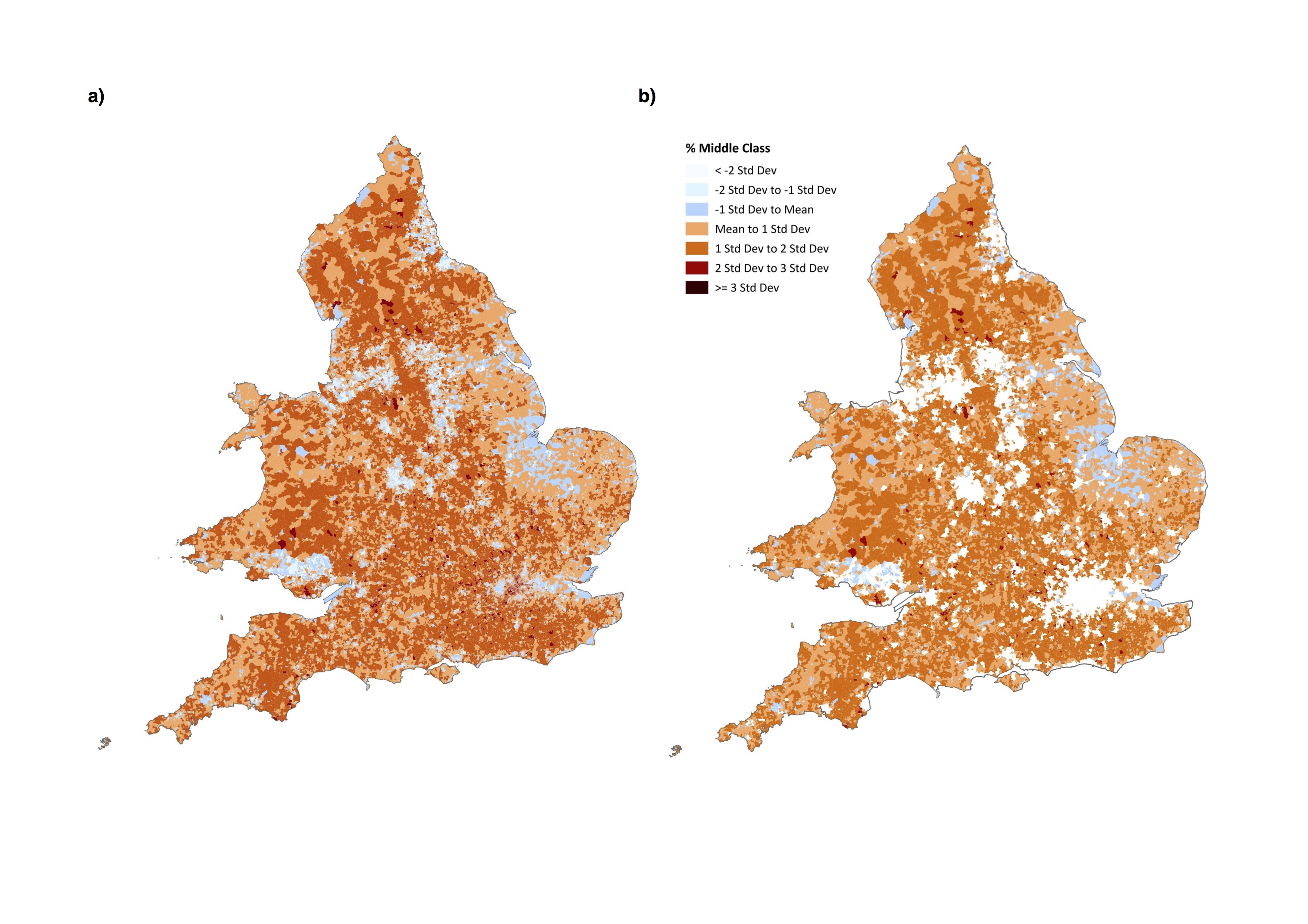

Supplement: Supplementary file 1 — Figure S1. The relative significance of the middle class in (a) England and Wales and (b) rural areas in England and Wales, 2011. Sources: Derived from Office for National Statistics, 2011 Census: Aggregate data (England and Wales) [computer file], UK Data Service Census Support (Downloaded from: http://infuse.mimas.ac.uk. Information licensed under the terms of the Open Government Licence [http://www.nationalarchives.gov.uk/doc/open‐government‐licence/version/2]); Digitised Boundary Data (England and Wales) [computer file], UK Data Service Census Support (Downloaded from: http://edina.ac.uk/census); Rural‐urban classification (2011) of output areas (2011) E + W (Downloaded from: https://geoportal.statistics.gov.uk). [file SORU-56-220-s001.jpg]

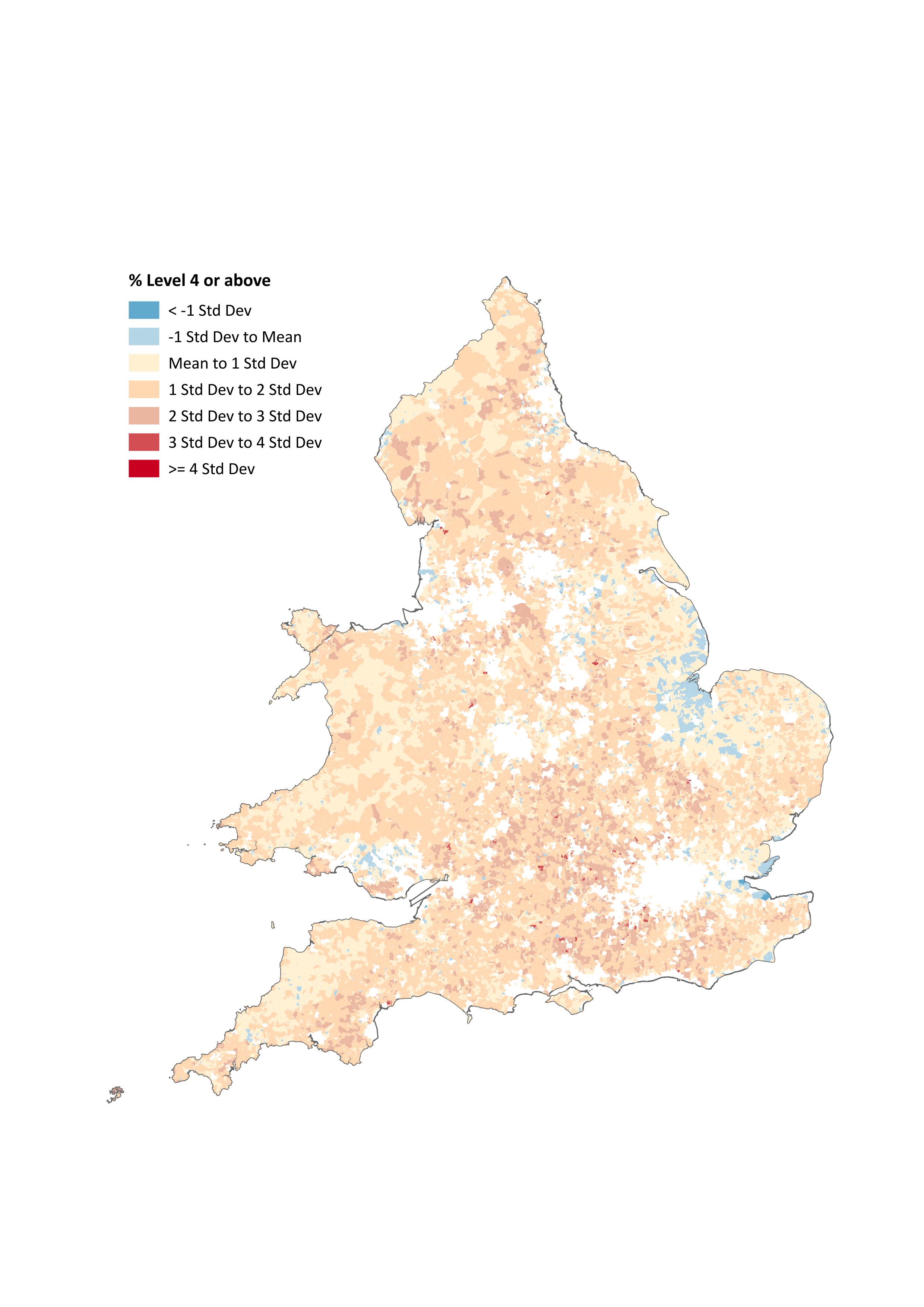

Supplement: Supplementary file 2 — Figure S2. Proportion of residents with Level 4 qualification within rural output areas in England and Wales. Sources: Derived from Office for National Statistics, 2011 Census: Aggregate data (England and Wales) [computer file], UK Data Service Census Support (Downloaded from: http://infuse.mimas.ac.uk. Information licensed under the terms of the Open Government Licence [http://www.nationalarchives.gov.uk/doc/open‐government‐licence/version/2]); Digitised Boundary Data (England and Wales) [computer file], UK Data Service Census Support (Downloaded from: http://edina.ac.uk/census); Rural‐urban classification (2011) of output areas (2011) E + W (Downloaded from: https://geoportal.statistics.gov.uk). [file SORU-56-220-s002.jpeg]
